# Poisons and Poisoning

**Published:** 1916-01-22

**Authors:** 


### POISONS AND POISONING.

### Some Legal Difficulties Explained.

Poisons and poisoning are subjects which interest both the medical profession and the public, and in their relations to crime they have necessarily, and on more than one occasion, engaged the attention of the Legislature. At the outset is the question of definition, and here science and law have alike put forth their powers. Equally, it may be said that neither the one nor the other has achieved an unqualified success. Probably, in the rough practical sense of the term, difficulty would not be admitted, and the man in the street is sure that he knows a poison when he sees one, just as he knows an elephant without attempting its definition. If pressed in relation to a poison he would probably say that it is a substance which is likely to destroy life when taken even in small quantity, and his fellows will hardly contradict him. But if, in cross-examination, he is asked, " What is a small quantity? " he will not easily escape his tormentor; and when brought to book by such a practical issue as, " Is alcohol a poison? " his answer is likely to vary according to his habits or prejudices. Manifestly, then, the popular use of the term will not afford a certain guide in a department where some accuracy of speech and of judgment is necessary.

### What is a Poison?

The severity of the puzzle to the scientific mind is illustrated in all the text-books which have toxicology for their subject-matter. Each new author attempts his own individual effort, and, having discussed the errors of his predecessors, promptly produces a definition which suffers a similar discipline from those who come after him. How, for instance, is it possible to range satisfactorily substances which in given conditions are useful remedies, and in other conditions quickly cause death? To say that strychnine is a nerve tonic is no doubt true, but common sense will not allow it to escape inclusion among the poisons. Is a dividing line in such circumstances possible? Again, suppose a considerable quantity of such a corrosive fluid as sulphuric acid be thrown over a man's face and body, the victim may quite possibly die from the injury and shock. But no one will admit that he was poisoned. Yet had the same fluid been poured down the man's throat, though death would be produced by exactly the same physiological machinery as that involved in the external application, there would be universal agreement that the man had been poisoned by sulphuric acid. In a vague way the popular belief is that all substances which are swallowed gain admission to the blood, and that such a substance as sulphuric acid may cause death in this fashion. But this, of course, is quite incorrect. The true statement is that death in such circumstances is due to shock caused by physical and chemical injury. When such injury is inflicted on the external surface of the body it would sound absurd to call the fatal issue death by poisoning. Strictly speaking, and were the same reasoning applied, death from swallowing sulphuric acid, therefore, ought not to be called by this term. Yet in practice it would be impossible to maintain such a conclusion. Here, indeed, is one of the main difficulties in framing a scientific definition of a poison. The definition has to include two groups of substances which, when they act fatally, produce death by entirely different methods; some cause local destructive effects on the parts to which they are directly applied, while others are only prejudicial when they are absorbed into the blood stream and are conveyed by this to remote organs. Regarding the latter group, there is an important practical consideration when one of their number is alleged to have been used for homicidal purposes. In trying to establish such a charge the prosecution can hardly be satisfied by proving the presence of the poison in the stomach of the victim; for so long as the poison remains in this situation it is not capable of producing a fatal result. Of course such a discovery might be, and probably would be, a highly suspicious circumstance, but it leaves open to the defence the suggestion that death was due, not to the poison, but to some other influence; and the proposal has even been made on the part of the prisoner that the poison might have been introduced into the stomach after death! It is to close these possible avenues of escape that great importance is placed in any charge of criminal poisoning upon the detection of the poison in the liver or other organs which can only be reached through the agency of the blood-stream. Once let this be proved, and it is then certain that the poison has reached a situation in which it has the opportunity to work its evil influence. For a similar reason the absence of poison from the stomach may be quite insufficient to negative a charge of poisoning, and this for the ample reason that there may have been time for its absorption, the actual fact of absorption being established by its discovery in the solid organs.

### A Definition Essential.

To come back to the question of definition, it is obvious that in some way or other the difficulty must be dealt with for legal purposes. Unless there is a clear and sufficient statement in answer to the question, " What is a poison? " regulations for the custody and sale of such substances are not possible, nor can a criminal be indicted on a charge of poisoning. It is in the two circumstances just suggested that the Legislature has been brought face to face with the problem, and it has found in each instance a special way of surmounting the difficulty. Taking the first of these two demands, it is obvious that substances so capable of mischief in the hands of careless or evil persons as are, say, strychnine and opium and prussic acid, cannot be allowed to be obtained by the first comer. Equally evident is it that their legitimate use as medicines, and in some instances their economic or commercial values, must be available where required. These two conditions the Legislature has had to satisfy. But at the outset, as already indicated, there appeared the difficulty of definition. How could an Act to regulate the sale of poisons be passed without a clear definition of the meaning of the word? The obvious way of escape was to proceed by schedule, in which all the substances to which the provisions of the Act were to apply should be duly named. This was the plan actually adopted. It may be said that two aims were intended. The first was the restriction of the sale of the substances named in the schedule to persons who were trained to appreciate their dangerous qualities. Hence it is the law of the land that (subject to a few exceptions) none of these scheduled "poisons" may be sold save by a qualified pharmacist. The second object to be secured was a warning to the purchaser of the dangerous nature of the substance he had purchased, and further, the provision of means by which the purchaser could be traced and identified should there be any ground for suspecting that he had used the "poison" for criminal purposes. Thus the Legislature decreed that every parcel or vessel containing the poison shall be duly labelled with the name of the substance, with the word "Poison" and with the name and address of the seller. In certain cases more than this is demanded. The schedule, as a matter of fact, is divided into two parts, and the conditions of sale just mentioned apply to all the substances mentioned in each of them. But in Part I. are named the specially dangerous "poisons," such, for example, as the poisonous vegetable alkaloids, corrosive sublimate, prussic acid, etc., and to the sale of each of these special provisions apply over and above those already described. It is necessary here that the purchaser be known to the seller or be introduced to him by some person known to both of them, that a register of the sale, with particulars as to the purpose for which the poison is required, be entered in a special book, and that the entry be attested by the signature of the purchaser, together with his address. This arrangement provides machinery by which the possession of a certain "poison" by a given individual can, if necessary, be proved, and this in connection with a charge of poisoning may obviously be of the very first importance. Thus, without attempting to define a "poison," the Legislature has made provisions which govern the sale of certain dangerous substances in such a fashion that the safety of the public is promoted and the detection of criminals facilitated.

### What is a Noxious Thing?

For the purposes which they have in view the above restrictions may be regarded as sufficient. But something more is clearly necessary if the ingenuity of the criminal poisoner is to be defeated, or at least if his crime is to be punished. It might well occur that such a person might employ for criminal purposes an agent other than those mentioned in the poisons schedule, and did this latter stand alone a charge of poisoning in such circumstances could only be framed with great difficulty. Science discovers new toxicological possibilities, and many substances which in ordinary phraseology would not be deemed poisons might well be given in an amount sufficient to produce harm or to destroy life. The evil intent would exist and might be successful, though it would be difficult or impossible to say in a narrow, scientific sense that the case was one of poisoning. To meet such possibilities, and to avoid all possible chances of a mere technical defence and the escape of a criminal, the Legislature again makes no attempt at definition, but labels as a criminal offence the administration or attempted administration of "a poison or other destructive or noxious thing." Thus no question can arise whether the substance employed is or is not a "poison" either in the popular or in the scientific meaning of this term. What is necessary to prove, beyond the fact of administration, is that the substance as administered actually did harm or was capable of doing harm, and that it was given with an evil intent. The law, in other words, does not say that certain substances are poisons and must not be administered, with the necessary implication that all others may be used at will. It asks whether the substance used is or is not "noxious," and whether it was or was not used from a criminal motive. When such manifestly "noxious" substances as strychnine or arsenic, for example, are in question the very nature of the substance proclaims the "evil intent," and this the more so that the criminal poisoner, to make sure of his purpose, usually employs relatively large amounts; or, using smaller amounts, he repeats the dose again and again. In such circumstances there is manifestly only one interpretation possible. The nature of the substance and the amount given proclaim "evil intent" beyond all question. But legal difficulties have arisen when the substance employed is not one that would be called a "poison" in the popular sense, or when, falling into this category, only very small doses have been employed. Here medical evidence of the possible or probable effects of the amount employed becomes of very great moment. For there is a definite ruling that, in order to establish an indictment charging a person with the administration of a "noxious" thing, it is necessary to prove that the substance in the dose administered or attempted to be administered was capable of proving "noxious." In this particular instance the substance concerned was cantharides, and there was no doubt that it was employed for a criminal purpose. But as the evidence showed that the amount administered was not sufficient to be capable of harming the victim, the poisoner escaped on the ground that the administration of a "noxious thing" implies administration in an amount capable of proving "noxious." Thus the issue is never an academic debate whether the "thing" is *noxious in itself*, but whether it was *noxious as administered*. These, briefly, are the fashions by which for legislative ends the difficulties of definition of the term "poison" are successfully obviated.

